# Knockdown of the bovine prion gene PRNP by RNA interference (RNAi) technology

**DOI:** 10.1186/1472-6750-7-44

**Published:** 2007-07-26

**Authors:** Shizuyo Sutou, Miho Kunishi, Toshiyuki Kudo, Pimprapar Wongsrikeao, Makoto Miyagishi, Takeshige Otoi

**Affiliations:** 1 School of Pharmacy, Shujitsu University, 1-6-1 Nishigawara, Okayama 703-8516, Japan; 2 Laboratory of Animal Reproduction and Biotechnology, Veterinary Sciences Yamaguch University, 1677-1 Yoshida, Yamaguchi, 753-8515, Japan; 3 Department of Surgery and Theriogenology, Faculty of Veterinary Medicine, Khonkaen University, 40000, Thailand; 4 21st Century Center of Excellence Program, Graduate School of Medicine, The University of Tokyo, Hongo 7-3-1, Bunkyo-ku, Tokyo 113-8655, Japan

## Abstract

**Background:**

Since prion gene-knockout mice do not contract prion diseases and animals in which production of prion protein (PrP) is reduced by half are resistant to the disease, we hypothesized that bovine animals with reduced PrP would be tolerant to BSE. Hence, attempts were made to produce bovine *PRNP* (b*PRNP*) that could be knocked down by RNA interference (RNAi) technology. Before an in vivo study, optimal conditions for knocking down b*PRNP* were determined in cultured mammalian cell systems. Factors examined included siRNA (short interfering RNA) expression plasmid vectors, target sites of *PRNP*, and lengths of siRNAs.

**Results:**

Four siRNA expression plasmid vectors were used: three harboring different cloning sites were driven by the human U6 promoter (hU6), and one by the human tRNA^Val ^promoter. Six target sites of bovine *PRNP *were designed using an algorithm. From 1 (22 mer) to 9 (19, 20, 21, 22, 23, 24, 25, 27, and 29 mer) siRNA expression vectors were constructed for each target site. As targets of siRNA, the entire b*PRNP *coding sequence was connected to the reporter gene of the fluorescent EGFP, or of firefly luciferase or *Renilla *luciferase. Target plasmid DNA was co-transfected with siRNA expression vector DNA into HeLaS3 cells, and fluorescence or luminescence was measured. The activities of siRNAs varied widely depending on the target sites, length of the siRNAs, and vectors used. Longer siRNAs were less effective, and 19 mer or 21 mer was generally optimal. Although 21 mer GGGGAGAACTTCACCGAAACT expressed by a hU6-driven plasmid with a *Bsp *MI cloning site was best under the present experimental conditions, the corresponding tRNA promoter-driven plasmid was almost equally useful. The effectiveness of this siRNA was confirmed by immunostaining and Western blotting.

**Conclusion:**

Four siRNA expression plasmid vectors, six target sites of b*PRNP*, and various lengths of siRNAs from 19 mer to 29 mer were examined to establish optimal conditions for knocking down of b*PRNP *in vitro. The most effective siRNA so far tested was 21 mer GGGGAGAACTTCACCGAAACT driven either by a hU6 or tRNA promoter, a finding that provides a basis for further studies in vivo.

## Background

Prion diseases are characterized by a prolonged latent period and a distinctive neuropathology that includes spongiform change, gliosis, neuronal loss, and the accumulation of an abnormal prion protein (PrP^Sc^), an isomer of the normal cellular prion protein (PrP^C^) encoded by the prion gene (*PRNP*), in affected brains. PrP^C^, a glycoprotein, is anchored to the outer surface of neurons and to a lesser extent of lymphocytes and other cells. The function of PrP^C ^is not known, but seems to be physiologically important because *PRNP *has been found in all animals examined (cattle, goats, hamsters, humans, mice, rats, sheep) as well as in the chicken. The conversion of PrP^C ^to PrP^Sc ^is believed to occur not as a result of viral or bacterial infection but as a result of interaction with exogenously introduced, self-replicating PrP^Sc ^or by a very rare spontaneous event according to the protein-only hypothesis [1].

Although data have been accumulated using BSE-infected mice [2] and *Prnp *knockout mice as well [3-5], the physiological role of PrP^C ^still remains to be clarified. From the pathological viewpoint, however, it is important that mice devoid of PrP^C ^are resistant to scrapie and fail to propagate prions [6-9] and that the introduction of PrP-encoding transgenes restores susceptibility to the disease [10]. *Prnp*0/+ mice, which have about half the normal level of PrP^C ^in their brains, show enhanced resistance to scrapie, as revealed by a significant delay in the onset and progression of clinical disease, while in wild-type animals, an increase in prion titer and PrP^Sc ^levels was followed within weeks by symptoms of scrapie and death [11]. These findings suggest that the production of BSE-resistant cattle would be possible by knocking down bovine *PRNP *(b*PRNP*) using RNA interference (RNAi) technology.

The injection of double-stranded RNA (dsRNA) into the cells of worms led to efficient sequence-specific gene silencing, referred to as RNAi [12]. This phenomenon also occurs in fly and plant cells, but not in mammalian cells. However, Elbashir et al. [13] demonstrated that 21~22-nt dsRNA with 2-nt 3' overhangs (short interfering RNA: siRNA) can induce sequence-specific gene silencing without non-specific inhibition of gene expression in cultured mammalian cells. siRNA expression systems using plasmid vectors are advantageous, because their use makes it possible to make transgenic animals, and the incorporation of one or a few plasmids into the nucleus would provide enough siRNA to induce RNAi. Soon after the discovery by Elbashir et al. [13] of the occurrence of RNAi in mammals, siRNA expression vector systems were developed [14-16]. The current understanding of the mechanisms of RNAi is as follows: dsRNA is digested to siRNA by the actions of Dicer, a family member of RNase III enzymes [17], and one strand of the siRNA unwound with the aid of ATP hydrolysis is incorporated into RISC (RNA-induced silencing complex) which has RNase activity [18]. mRNA with the sequence complementary to the siRNA is cleaved by RISC, knocking-down the gene expression of a specified mRNA (Dykxhoom et al., review [19]). Here we present optimal conditions, including siRNA expression promoters, target sites, and lengths of siRNAs, for knocking down b*PRNP*.

## Results

### Target plasmids with the insertion of a stop codon between reporter and target genes

In previous experiments, a full-length mouse *Prnp *gene with ATG (762 bp) or without ATG (759 bp) and three other fragments (645, 486, and 306 bp from the stop codon) were ligated in-frame into pDsRed2-C1. Cells transfected with the full-length construct and the plasmid with the 486-bp fragment did not show fluorescence, but cells transfected with the plasmids containing the 645-bp and 306-bp fragments emitted fluorescence, indicating that in-frame inserts might stop the production of the fluorescent reporter depending on the sequence. When the full-length b*PRNP *was inserted into pEGFP-C1 downstream from the EGFP stop codon, cells transfected with the construct emitted fluorescence. Therefore, a stop codon was inserted between the reporter and b*PRNP *in all the target plasmids, all of which were found to be effective in producing fluorescence in transfected cells (Table 1).

**Table 1:** Characteristics of target vectors

Vector	Promoter 1	Reporter 1	Target	Promoter 2	Reporter 2	Target
pEGFP-bPrP	CMV	EGFP	b*PRNP*			
pGL3-bPrP	SV40	Fire-fly luciferase	b*PRNP*			
pFluc-bPrP-Rluc	SV40	Fire-fly luciferase	b*PRNP*	HSV-TK	Renilla luciferase*	
pFluc-Rluc-bPrP	SV40	Fire-fly luciferase*		HSV-TK	Renilla luciferase	b*PRNP*
pTKFluc-bPrP-Rluc	HSV-TK	Fire fly luciferase	b*PRNP*	HSV-TK	Renilla luciferase*	

*, used as the internal control.

### Dose-response relationship between fluorescence and plasmid DNA

When the dose-response relationship was examined using pEGFP-bPrP, linearity of EGFP fluorescence was obtained below 500 ng/well; a total of 400 ng/well or less was therefore used in subsequent experiments.

### Comparison of sensitivity of fluorescence versus luminescence and lengths of siRNAs

**Figure 1:**
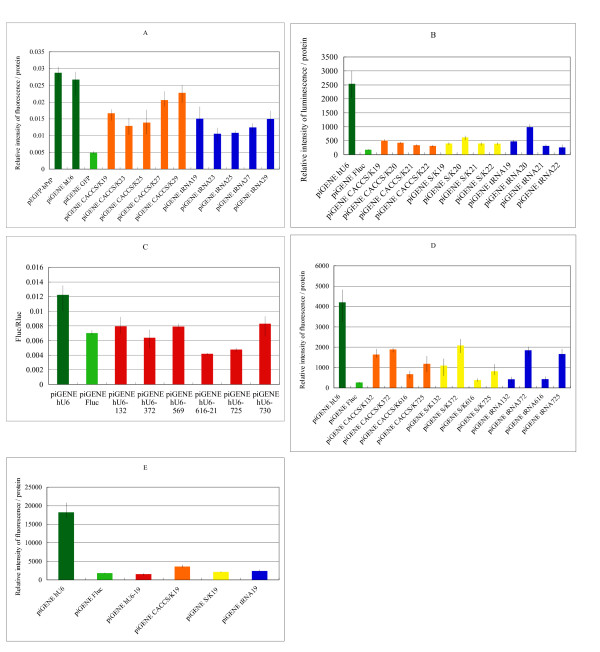
**Effects of siRNA expression vectors on b*PRNP***. A and B, effects of different lengths of siRNAs expressed by different vectors. A: piGENE hU6-EGFP was a positive control siRNA vector for EGFP. Numerals after vectors indicate the lengths of siRNA; 19, 23, 25, 27, and 29 correspond to No. 6, 10, 12, 13, and 14 in Table 2, respectively. B: piGENE Fluc is a positive control siRNA vector against firefly luciferase. Numerals after vectors indicate the lengths of siRNA; 19, 20, 21, and 22 correspond to No. 6, 7, 8, and 9 in Table 2, respectively. C and D, effects of different target sites and expression vectors on siRNA activities. C: numerals after vectors indicate the start sites of siRNA; 132, 372, 616, and 725 correspond to No. 2, 4, 9, and 16 in Table 2, respectively. D: as for numerals after vectors, see the legend to C. E: comparison of vector activities. Vectors expressed 19 mer, and the sequence corresponds to No. 6 in Table 2.

When the sensitivity of the fluorescent reporter of pEGFP-bPrP (Fig. 1A) and the luminescent reporter of pGL3-bPrP (Fig. 1B) was compared, detection of the EGFP fluorescence was found to be less sensitive than detection of the luminescence produced by firefly luciferase. This may be due to the comparatively long life of EGFP protein. Fig. 1A also shows that shorter siRNAs (around 23 mer) were more effective in silensing b*PRNP *expression than longer ones (27 and 29 mer), and that piGENE tRNA was more effective than piGENE CACC-S/K. Fig. 1B compares a narrow range of lengths of siRNAs expressed by three plasmid vectors. On the whole, 21 or 22 mer was the most effective silencer, and the differences between them were minor.

### Vectors harboring internal control emitters

To determine the effectiveness of siRNAs, measurements of reporter protein levels were made using fluorescence or luminescence reporter assays (Fig. 1A and 1B). Three vectors in which an internal control was integrated into a single target vector, pFluc-bPrP-Rluc, pFluc-Rluc-bPrP, and pTKFluc-bPrP-Rluc, were constructed (Table 1). Since the results obtained with pTKFluc-bPrP-Rluc were almost the same as those for pFluc-Rluc-bPrP and pTKFluc-bPrP-Rluc, the results for pFluc-bPrP-Rluc are shown in Fig. 1C, in which effective target sites are compared (see next section). *Renilla* luminescence was always much stronger than firefly luminescence driven by either the SV40 or HCV-TK promoter. Although the sensitivity of siRNA effects detected using these double emitter vectors was low compared with that of a single emitter such as pGL3-bPrP, the relative effectiveness of siRNA was reproducible. Therefore, these vectors appeared to be useful for assaying the relative activity.

### Effective target sites

Six siRNA target sites were predicted in the b*PRNP *sequence by using an algorithm [20] (Nos. 1, 3, 5, 6, 15 and 17, Table 2). Six different 22-nt targets (Nos. 2, 4, 5, 8, 16 and 18, Table 2), except for No. 8, where a 21 mer was used, were compared using a single expression vector, piGENE hU6 (Fig. 1C). The target vector was pFluc-bPrP-Rluc and the sensitivity was quite low, as described previously, but relative activities could be determined. No. 8 with the sequence 5'-GGGGAGAACTTCACCGAAACT-3' was the best silencer. Fig. 1D shows differences in siRNA activities examined using combinations of four target sites of 22 nt (Nos. 2, 4, 9, and 16, Table 2) and three expression vectors (piGENE CACC-S/K, piGENE S/K, and piGENE tRNA. Table 3). As for vectors, piGENE S/K was the best, followed by piGENE tRNA; and piGENE CACC-S/K was almost always the worst. As for target sites, sequence No. 9, the 22 mer version of No. 8 (21 mer), was the best and No. 4 was the worst using all three vectors. Differences between 21 mer and 22 mer were minimal in the three vectors (Fig. 1D).

**Table 2:** Target sequences examined

No.	Position^a^	sequence	nt	Constructed vectors (○)^b^				Value^c^
				hU6	CACC-S/K	S/K	tRNA	
					
1	132–150	GGGCAGTCCTGGAGGCAAC	19	○	○	○	○	0.779
2	132–153	GGGCAGTCCTGGAGGCAACCGT	22	○	○	○	○	
3	372–390	AGGAGCTGCTGCAGCTGGA	19	○	○	○	○	0.775
4	372–393	AGGAGCTGCTGCAGCTGGAGCA	22	○	○	○	○	
5	569–591	GTGTCAATATCACAGTCAAGGA	22	○				0.757
6	616–634	GGGGAGAACTTCACCGAAA	19	○	○	○	○	0.852
7	616–635	GGGGAGAACTTCACCGAAAC	20	○	○	○	○	
8	616–636	GGGGAGAACTTCACCGAAACT	21	○	○	○	○	
9	616–637	GGGGAGAACTTCACCGAAACTG	22	○	○	○	○	
10	616–634	GGGGAGAACTTCACCGAAACTGA	23	○	○	○	○	
11	616–634	GGGGAGAACTTCACCGAAACTGAT	24	○				
12	616–634	GGGGAGAACTTCACCGAAACTGACA	25	○	○	○	○	
13	616–634	GGGGAGAACTTCACCGAAACTGACATC	27	○	○	○	○	
14	616–634	GGGGAGAACTTCACCGAAACTGACATCAA	29	○	○	○	○	
15	725–743	GTGTGATCCTCTTCTCTTC	19	○	○	○	○	0.772
16	725–746	GTGTGATCCTCTTCTCTTCCCC	22	○	○	○	○	
17	730–748	ATCCTCTTCTCTTCCCCTC	19	○				0.752
18	730–751	ATCCTCTTCTCTTCCCCTCCTG	22	○				

a, Position 1 starts from A of ATG, the first codon of b*PRNP*. b, hU6, CC-S/K, S/K, and tRNA indicate piGENE hU6, piGENE CACC-S/K, piGENE S/K, and piGENE tRNA, respectively (Table 3). Open circles designate construction of vectors. c, Values were obtained from the prediction algorithm [20].

**Table 3:** Structure of cloning sites of siRNA expression vectors

Vector	Promoter	Sequence of cloning site
piGENE hU6	hU6	CACCGTGAGCAGGTGTAAAGCCACCATGGAAGACACCTGCCAAC *TTTTTT*CAATTGGTCGACCTGCAGGCATGCAAGCTT^a^
piGENE CACC-S/K	hU6	CACCGAGCTCAGACTCGATATCGGTACC^b^
piGENE S/K	hU6	GAGCTCAGACTCGATATCGGTACC^c^
piGENE tRNA	tRNA	GAGCTCCAGATCTAATGCGGCCGCTTAGGTACCATAGATATC*TTTTTTT*CTGCAGGCATGCAAGCTT^d^

a: Three *Bsp *MI sites are underlined. b and c: *Sac *I, *Bgl *II, *Eco *RV, and *Kpn *I sites are underlined. d: *Sac *I, *Bgl *II, *Not *I, *Kpn *I, *Eco *RV, *Pst *I, and *Hind *III sites are underlined. T repeats, RNA pol III terminator signal, are italicized.

### Comparison of siRNA expression vectors

The structure of the cloning sites of the siRNA expression vectors is shown in Table 3. Fig 1E compares the effectiveness of the siRNA expression vectors. Fig. 1E shows a typical result, and the order of effectiveness was almost always piGENE hU6, piGENE S/K, piGENE tRNA, and piGENE CACC-S/K. The difference between piGENE hU6 and piGENE S/K was minimal and sometimes the order of these two was reversed.

### b*PRNP* silencing as revealed by immunostaining

**Figure 2:**
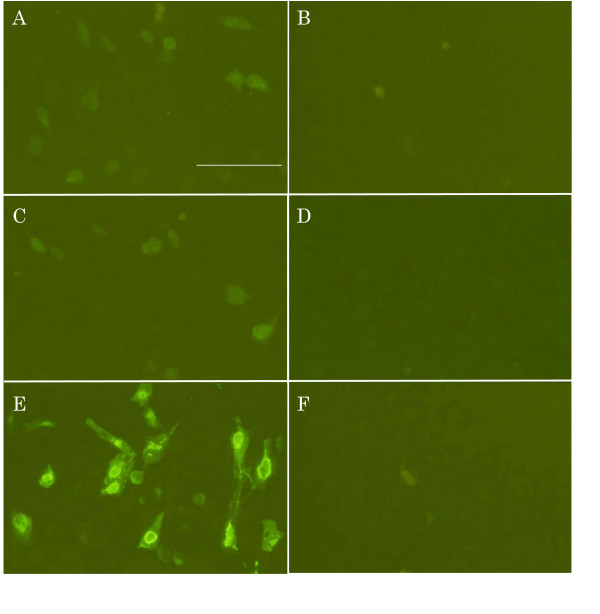
**Silencing of b *PRNP *by siRNA as revealed by immunostaining**. HeLaS3 cells were co-transfected with pbPrP- FLAG DNA and the control vector DNA of piGENE tRNA (A) or siRNA expression vector DNA of piGENE tRNA-616-21, the sequence of which is No. 8 in Table 2 (B). Cells were stained with SAF32 antibody. HeLaS3 cells were co-transfected with pbPrP- FLAG DNA and the control vector DNA of piGENE hU6 (C) or siRNA expression vector DNA of piGENE hU6-616-21(D). Cells were stained with P6488 antibody. HeLaS3 cells were co-transfected with pshort-bPrP- FLAG DNA and the control vector DNA of piGENE hU6 (E) or siRNA expression vector DNA of piGENE hU6-616-21 (F). Cells were stained with P6488 antibody.

Full-length PrP^C ^and truncated PrP^C ^as targets and three antibodies, SAF32, P6488, and anti-FLAG, recognizing different regions, the upstream, mid-part, and C-terminal tag, respectively, were used for immunostaining experiments. Full-length PrP^C ^could be detected equally using the three antibodies; the fluorescence signal intensities, which were moderate, were almost the same (Fig. 2A and 2C) among the three. The number of fluorescent cells depended on the amount of target DNA applied. When 800 ng/well was applied, approximately 50% of cells were fluorescence-positive, and when 200 ng/well was used, 20–30% were positive. When target DNA was co-transfected with siRNA expression vector DNA, almost no fluorescence was detected (Fig. 2B). This was true for both hU6 and tRNA promoter-driven vectors (Fig. 2B and 2D). Truncated PrP^C ^could be detected using P6488 (Fig. 2E) and anti-FLAG antibody. As expected, SAF32 could not detect truncated PrP^C^, which lacks the N-terminal region (not shown). The intensity of fluorescence was much stronger than that of full-length PrP^C ^(compare Fig. 2E with A and C). Perinuclear regions were strongly stained (Fig. 2E). The fluorescence almost disappeared when target DNA was co-transfected with siRNA expression plasmid DNA derived from either piGENE hU6 (Fig. 2F) or piGENE tRNA, indicating the effectiveness of siRNA.

### b*PRNP* silencing as revealed by Western blotting

**Figure 3:**
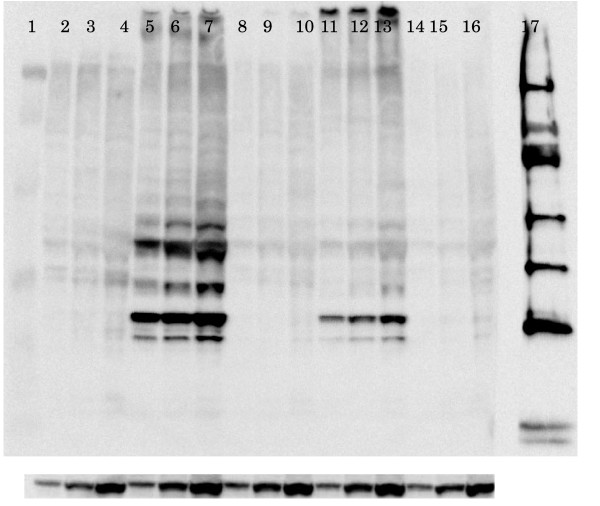
**Silencing of b *PRNP *by siRNA as revealed by Western blot analysis**. Top, protein size markers (Bio-Rad, lane 1), proteins from HeLaS3 cells (lanes 2–4), proteins from cells co-transfected with pbPrP- FLAG DNA and the control vector DNA of piGENE hU6 (lanes 5–7) or siRNA expression vector DNA of piGENE hU6-616-21, the sequence of which is No. 8 in Table 2 (lanes 8–10), proteins from cells co-transfected with pbPrP- FLAG DNA and the control vector DNA of piGENE tRNA (lanes 11–13) or siRNA expression vector DNA of piGENE tRNA-616-21(lanes 14–16), and protein size markers (150, 100, 75, 35, 25, and 15 kDa from the top, GE Healthcare). Amounts of proteins applied were 1.25 (lanes 2, 5, 8, 11, 14), 2.5 (lanes 3, 6, 9, 12, 15), and 5.0 (lanes 4, 7, 10, 13, 16) μg/lane. The gel was stained with SAF32 antibody. Bottom, actin staining.

When a full-length PrP^C ^was transiently expressed and detected using the antibody SAF32, 25- to 30-kDa bands were detected, with the 26-kDa band being most intense. When target DNA was co-transfected with siRNA expression vector DNA, almost no bands were detected (Fig 3), indicating the effectiveness of siRNA. This was true for both hU6 (Fig 3, lanes 8–10) and tRNA (Fig 3, lanes14–16) promoter-driven vectors. The bands in lanes 5–7 were darker than those in lanes 11–13. Sonication of the samples in lanes 11–13 seemed to have been insufficient, because intense bands were seen at the sites of sample application. The major band was 26 kDa, non-glycosylated PrP^C^. Glycosylated PrP^C ^appeared at around 32 kDa, as can be clearly seen in lanes 5–7, and very faintly seen in lanes 11–13. The nature of the strong bands at around 40 kDa in lanes 5–7 is not known. Truncated PrP^C ^could not be detected using SAF32, as expected, but could be detected using P6488 antibody (data not shown).

## Discussion

The hU6 promoter has been widely used to drive siRNA expression in plasmid vectors, but tRNA promoter-driven vectors have rarely been used; therefore, in this study piGENE tRNA was used for comparison with piGENE hU6. These two vectors have different cloning sites, i.e., piGENE hU6 has a *Bsp *MI cloning site, while the piGENE tRNA has a *Sac *I, *Bgl *II, *Not *I, *Kpn *I, and *Eco *RV cloning site, (Table 3). The instructions of the manufacturer are also different: 19 to 22 mer is reccomended for piGENE hU6 and less than or close to 30 mer for piGENE tRNA. siRNA activities are affected by the promoters, siRNA lengths, and cloning site structures. To compare the effectiveness of piGENE hU6 and piGENE tRNA, two other siRNA-expression vectors were constructed; these were piGENE CACC-S/K and piGENE S/K, which had a common cloning site consisting of *Sac *I, *Bgl *II, *Eco *RV, and *Kpn *I recognition sequences. Using the *Sac *I and *Kpn *I sites was convenient because the same constructs could be employed as cassettes for piGENE CACC-S/K, piGENE S/K, and piGENE tRNA (Table 3). CACC was introduced into piGENE CACC-S/K because the natural human U6 promoter habors a G just after CACC, which enhances transcription [20].

siRNA activities can depend on the target genes and their sequences, and therefore definite and universal conclusions cannot be made. However, the present data indicate that a shorter siRNA (around 19 to 22 mer rather than 30 mer) is better for piGENE tRNA. As a whole, piGENE hU6 showed higher levels of siRNA expression activity than piGENE tRNA. piGENE hU6 and piGENE S/K had almost the same levels of activity, but the former seemed to be slightly better. The *Sac *I recognition site is GAGCTC and the insertion of DNA for siRNA expression occurred just after this site. On the other hand, the inserts come after CACCGAGCTC in piGENE CACC-S/K, the siRNA transcription of which might start from G, A, or G in the *Sac *I recognition site, and Dicer counts base numbers from these starting sites. An unexpected early start to transcription would leave several bases at the 3' end behind and lead to a shortage of matching bases to target mRNAs, lessening siRNA acrtivity.

As for the length of siRNA, 22 mer was generally the best (Fig. 1B), although 21 mer sometimes showed similar or better activity. However, 20 mer usually showed less activity than 19 mer, 21 mer or 22 mer (Fig. 1B). The reasons for this are not known, but Dicer's mechanisms of action might be involved.

The target sequence No. 6, 5'-GGGGAGAACTTCACCGAAA-3', achieved the highest score among the six targets (Table 2). Principles for the prediction of a favorable siRNA have been proposed [21,22]. One of the key factors in RNAi is the assembly of RISC, which mediates target RNA cleavage. The sense and anti-sense strands of an siRNA duplex are not equally eligible for assembly into RISC. Both the absolute stability and relative stability of the base pairs at the 5' end of siRNA seem to determine which strand takes part in the RNAi pathway. Given that in the conventional way of writing DNA sequences, the sense strand is the upper one and the anti-sense strand is the lower one, the left end should be tight and the right end should be loose for the anti-sense strand to be incorporated into RISC. In such an analysis, No. 6 has a tight left and a loose right end, and thus it is theoretically predicted to be effective, and was in fact found to be effective. This tightness and looseness do not always determine the effectiveness of RNAi, but are important factors.

The purpose of the present study was to knockdown b*PRNP *using RNAi technology. We used, however, an siRNA expression vector driven by a hU6 promoter and human HeLaS3 cells in an in vitro system. Is the hU6 promoter active in bovine cells? Recently, we [23] cloned chicken U6 promoters and examined their activity in chick cells using the hU6 promoter as a comparative control. Chicken and human promoters showed almost the same level of activity, although the activity levels were not very high. Lambeth et al. [24] cloned a bovine U6 promoter and examined its activity in MDBK (Madin Darby Bovine Kidney) and Vero (African Green monkey kidney) cells together with the mouse U6 promoter. The U6s of both species gave almost identical results, suggesting that the hU6 promoter might also be active in different mammalian cells. Indeed, our experiments showed that siRNA driven by hU6 was active in bovine primary cultured cells (data not shown).

## Conclusion

To produce b*PRNP*-knocked down cattle using RNA interference (RNAi) technology, optimal conditions for knocking down were first investigated in vitro. Four siRNA expression plasmid vectors, six target sites of b*PRNP*, and various lengths of siRNA from 19 mer to 29 mer were examined. As a target, the b*PRNP *coding sequence was connected to the reporter of the fluorescent EGFP, firefly luciferase, or *Renilla *luciferase gene. When target plasmid DNA was co-transfected with siRNA expression vector DNA into HeLaS3 cells, and fluorescence or luminescence was measured, siRNA of 21 mer GGGGAGAACTTCACCGAAACT expressed by a hU6- or tRNA-driven plasmid gave the best knockdown result under the present experimental conditions. The effectiveness of this siRNA was confirmed by immunostaining and Western blotting. These data provide a basis for further studies in vivo.

## Methods

### Cloning of bovine prion gene *PRNP*

b*PRNP *consists of three exons, with the open reading frame (ORF) being located in exon 3 [25,26] (see also GeneBank Accession No. D10612). An ORF consisting of 792 bp encoding 264 amino acid residues was amplified by PCR with a pair of primers, one with a *Bgl *II site at the terminus and the other with a *Hind *III site. The PCR product was inserted into a TOPO vector (Invitrogen, Carlsbad, CA).

### *PRNP* target vectors

pEGFP-C1, which harbors a green fluorescent protein gene driven by the CMV promoter, was purchased from Becton, Dickinson and Company, Japan (Tokyo, Japan). The multiple cloning sites of the vector were changed to *Bsr *GI-*Bsp *EI-*Spe *I-*Bgl *II-*Sal *I-*Afl *II-*Spl *I-*Cla *I-*Apa *I-*Sma *I/*Xma *I-*Bam *I-*Xba *I-*Bcl *I. This vector has an in-frame stop codon, TAG, in the *Spe *I recognition site (ACTAGT), which deletes the translation of inserts cloned downstream of the *Bgl *II site so as not to interfere with the intensity of fluorescence. pGL3 control and pRL-TK, which harbor the firefly luciferase gene (Fluc) driven by the SV40 promoter and the *Renilla* luciferase gene (Rluc) driven by the HSV-TK promoter, respectively, were purchased from Promega KK (Tokyo, Japan). The multiple cloning sites and *Hind *III site of the pGL3 control were removed and a new multiple cloning site, *Xba *I, *Aat *II, *Bgl *II, *Sal *I, *Pst *I, *Apa *I, *Sma *I/*Xma *I, *Spe *I, *Afl *II, and *Spl *I, was introduced just after the stop codon at the end of Fluc. The b*PRNP *gene was cloned between the *Bgl *II and *Xma *I sites. To use either Fluc or Rluc as an internal control, a combination vector, pRL-TK/SV-FL, was constructed by inserting the *Bgl *II and *Bam *HI fragment of the pGL3 control, which encodes the SV40 promoter and Fluc, into the *Bam *HI site of pRL-TK. Three b*PRNP *target plasmids were constructed from pRL-TK/SV-FL (Table 1).

### PrP^C^-FLAG and truncated PrP^C^-FLAG vectors

For immunostaining and Western blot analyses, a FLAG-tagged PrP^C ^expression vector was constructed by introducing the full-length b*PRNP *into the vector pFLAG-CMV-5 (Sigma-Aldrich, St. Louis, MO) between the *Bgl *II and *Sal *I sites. This vector, pbPrP- FLAG, was expected to produce a protein consisting of PrP^C ^(264 amino acid residues) connected with FLAG (8 amino acid residues). Since antibodies to detect PrP^C ^might hinder the identification of PrP^C ^because of similarity in the molecular size, a truncated pPrP-FLAG was also constructed by removing the N-terminal region between *Bgl *II and *Pvu *II from PrP^C^-FLAG. This pshort-bPrP- FLAG vector was expected to produce a protein consisting of the C-terminal PrP^C ^(145 amino acid residues) connected with FLAG (8 amino acid residues).

### Selection of favorable target sites and construction of hairpin-type siRNA

Six target sites for siRNA were predicted using an algorithm developed by Taira and Miyagishi [20]. Some derivatives with lengths different from the original six sites were constructed (Table 2). The hairpin loop sequence for these was GTGTGCTGTCC. A few mutations in the sense strand were introduced in all constructs to avoid plasmid instability and difficulty in sequencing.

### siRNA expression vectors

piGENE hU6 [14] has the human U6 promoter and a *Bsp *MI cloning site. piGENE tRNA Pur [27] has the human tRNA^Val ^promoter and a multiple cloning site, *Sac *I/*Bgl *II/*Not *I/*Kpn *I/*Eco *RV. Both were obtained from iGENE Therapeutics, Inc. (Tsukuba, Japan). Two vectors modified at the cloning site were made from piGENE hU6. One had a *Sac *I/*Kpn *I cloning site and the other a naturally occurring CACC sequence just before the *Sac *I/*Kpn *cloning site (Table 3).

### Cell culture and transfection

HeLaS3 cells were cultured in Dulbecco's modified Eagle's medium (Sigma, St. Louis, MO) supplemented with 10% fetal calf serum. Cells (0.75 × 10^5^/well) were plated in a 24-well plate and, a day after plating, transfected with plasmid DNA. A typical treatment consisted of 200 *μ*g of target vector DNA and 200 *μ*g of siRNA expression vector DNA. Transfection was performed using Lipofectamine 2000 (Invitrogen, Carlsbad, CA) according to the manufacturer's instructions.

### RNAi assays

Cells transfected with fluorescent-protein-expressing plasmid DNA were examined under a fluorescence microscope (Olympus IX71, Tokyo). To determine the intensity of fluorescence quantitatively, cells were lysed with 100 *μ*L of lysis buffer (Promega, Madison, WI) and aliquots (80 *μ*L) were analyzed using Fluoroskan Ascent FL (Thermo Labosystems, Helsinki, Finland). To determine the amount of protein, aliquots (5 *μ*L) were mixed with 200 *μ*L of 5-fold diluted reagent of the Bio-Rad Protein Assay (Bio-Rad Laboratories-Inc, Hercules, CA), and the absorption was measured using Multiskan Ascent (Thermo Labosystems). Comparisons were made on the basis of fluorescence per *μ*g protein. To measure luminescence, a Dual-luciferase Reporter Assay System (Promega) was used. The detector was a Sirius Luminometer (Berthold Detection Systems, Pforzheim, Germany). In this case, the intensity of the target (Fluc) was divided by that of the internal control (Rluc) or vice versa and the ratios were used for comparison.

### Immunostaining of PrP^C^ and truncated PrP^C^

HeLaS3 cells (4 × 10^4^/well, 24-well plate) were plated and, after 24 h of culturing, pbPrP- FLAG DNA or pshort-bPrP- FLAG DNA (200 ng) and siRNA expression vector DNA or control vector DNA (200 ng) were co-transfected for 4 h. After 24 h, cells were fixed with acetone-methanol (1:1) and washed with TBS (Tris buffered saline) for 10 min 3 times. After being treated with TBST (TBS with 0.05% Triton X) for 10 min and then washed with TBS once, cells were blocked with 10% BSA/TBS for 1 h with shaking. After a brief wash with TBS, cells were stained with a primary antibody for 1 h. The antibody was anti-PrP^C ^or anti-FLAG antibody diluted 2000 to 5000 fold. After another brief wash with TBS, cells were blocked with 3% BSA/TBS for 1 h, and then stained with a secondary antibody (rabbit anti-mouse-IgG-FITC (SIGMA) diluted 10000 fold) for 1 h. After three washes with TBST, cells covered with TBS were examined under a fluorescence microscope (Olympus IX71, Tokyo). One anti-PrP^C ^antibody was SIGMA P6488 (SIGMA; host, mouse; isotype, IgG_1_), which had been raised against a synthetic peptide corresponding to amino acid residues 146–159 of bovine PrP^C^. Bovine PrP^C ^was identified as a protein of 33–35 kDa that was immunoreactive with P6488, which also can detect human, sheep, deer, and elk PrP^C^. Another anti-PrP^C ^antibody was SAF32 (SPI Bio; host, mouse; isotype, IgG_2b_), which recognizes the octo-repeat region located in the N-terminal part of PrP and cross-reacts with bovine, human, hamster, mouse, and ovine PrP^C^. Bovine PrP^C ^and the truncated PrP^C ^were also detected using the anti-FLAG antibody M2 (isotype, IgG_1_) which was one of the components of SIGMA's kit (Carboxy-terminal FLAG mammalian transient expression kit).

### Western blot analysis

HeLaS3 cells (7 × 10^5^) were plated in a plastic dish 60 mm in diameter. After 24 h, cells were co-transfected with pbPrP- FLAG DNA or pshort-bPrP- FLAG (4 *μ*g) for 4 h. Cells were washed and cultured in fresh medium. After 24 h, they were washed with PBS once and collected in 100 *μ*L of TBS with a rubber policeman. The cells were lysed by sonication for 3 min on ice. An equal volume of Laemmli sample buffer (Bio-Rad) containing 5% mercaptoethanol was added to the cell lysate, and the mixture was boiled for 5 min. Samples were subjected to SDS-PAGE using the following conditions: electrophoresis buffer, 1 × Tris-glycine-SDS (10 fold dilution of 10 × buffer, Bio-Rad) containing 5% methanol; gel, SuperSep™ 10–20% (Wako Pure Chemical Industries, Inc., Osaka); markers, ECL DualVeu Western blotting markers (GE Health Care, Piscataway, NJ); and electrophoresis conditions, 20 mA for 80 min. Proteins were transferred to a Fluorotrans^® ^sheet (Nippon Genetics, Tokyo) in a Mini Transblot Cell (Bio-Rad) at 100 V for 60 min. The membrane was blocked with 5% skim milk/TBST for 1 h at room temperature. After being washed with TBST for 10 min 3 times, the membrane was stained with the antibody SAF32 (diluted 1/5000 with TBST) for 1 h with shaking. After 3 washes with TBST for 10 min each time, the membrane was stained with a secondary antibody, Anti-mouse IgG-HRP conjugate (idiluted 1/10000 with TBST, ECL Plus Western blotting detection kit, GE Healthcare), for 1 h with shaking. After 3 more washes with TBST for 10 min each time, the membrane was treated with a 40:1 mix of ELC Plus A:B(iWestern blotting detection kit) for 5 min. Photos were taken using a LAS-1000 (Fuji) with 30 sec to 2 min of exposure.

## Authors' contributions

SS, MK, and PW carried out all experiments and SS drafted the manuscript. MM predicted target sites. TK and TO participated in the planning, design, and coordination of the research. All authors read and approved the final manuscript.
